# Impaired peripheral mononuclear cell metabolism in patients at risk of developing sepsis: A cohort study

**DOI:** 10.2478/jccm-2026-0010

**Published:** 2026-01-30

**Authors:** Velma Herwanto, Ya Wang, Maryam Shojaei, Alamgir Khan, Kevin Lai, Amith Shetty, Stephen Huang, Tracy Chew, Sally Teoh, Marek Nalos, Mandira Chakraborty, Anthony S McLean, Benjamin M P Tang

**Affiliations:** Universitas Tarumanagara, Faculty of Medicine, Jakarta, Indonesia; Department of Intensive Care Medicine, Nepean Hospital, Kingswood, NSW, Australia; Australian Proteome Analysis Facility, Macquarie University,NSW, Australia; Department of Emergency Medicine, Westmead Hospital,NSW, Australia; Sydney Informatics Hub, The University of Sydney,NSW, Australia

**Keywords:** cellular metabolism, oxidative stress, peripheral blood mononuclear cells, sepsis, uncomplicated infection

## Abstract

**Introduction:**

Dysregulated immune responses are central to progression of sepsis and closely associated with impaired cellular metabolism. However, most existing studies have focused on late-stage sepsis, leaving metabolic alterations during earlier stages of infection poorly characterised. This study aimed to determine whether immune cell metabolic impairment is already present during uncomplicated infection, prior to the development of sepsis, and to evaluate its potential as an early indicator of immune dysfunction and risk of progression.

**Materials and methods:**

Forty patients with sepsis (fulfilling Sepsis-3 criteria) and 27 patients with uncomplicated infection were recruited from the emergency department along with 20 healthy volunteers. Whole blood samples were collected to assess gene expression, cytokine levels, and cellular metabolic functions, including mitochondrial respiration, oxidative stress, and apoptosis in immune cells.

**Results:**

Mitochondrial respiration was significantly impaired in immune cells from both uncomplicated infection and sepsis patients compared with healthy controls (p < 0.05), with more pronounced impairment in established sepsis. Downregulation of BCL2 and BBC3 gene expression was observed in sepsis patients (p < 0.05), but not in uncomplicated infection, potentially contributing to differences in the severity of metabolic impairment. Impaired mitochondrial respiration was significantly associated with increased mitochondrial oxidative stress (p < 0.05), which was elevated in uncomplicated infection and further increased in sepsis. Oxidative stress levels also correlated with tumour necrosis factor-α (r = 0.330) and the expression of CYCS, TP53, SLC25A24, and TSPO (rs = −0.4926, −0.4422, 0.4382, and 0.4835, respectively). Despite these metabolic alterations, no significant differences in immune cell apoptosis were observed between uncomplicated infection and sepsis patients.

**Conclusions:**

Immune cell metabolic dysfunction is present in patients with uncomplicated infection before the clinical onset of sepsis. Early mitochondrial dysfunction and oxidative stress may represent promising targets for further investigation as early biomarkers of immune dysfunction and sepsis risk.

## Introduction

Sepsis is one of the most significant disease burdens in the world [1]. A better understanding of sepsis disease mechanism is urgently needed to facilitate development of new therapies. Several pathophysiological mechanisms are involved, including endothelial dysfunction, coagulopathy and dysregulated immune response [2]. Among these, dysregulated immune response is thought to play the most critical role in sepsis. The dysregulated immune response in sepsis is characterized by impaired innate and adaptive immune responses; both of which have been shown to strongly correlate with poor patient outcomes [3, 4].

It has been demonstrated that impaired cellular metabolism contributes to the dysregulated immune response in sepsis. The impaired cellular metabolism includes reduced cellular oxygen utilization and reduced adenosine triphosphate (ATP) production [5, 6]. A number of studies in sepsis patients have confirmed that these impairments are associated with increased organ failures and reduced survival [7, 8].

Recent studies sought to identify defective pathways that are associated with impaired cellular metabolism in peripheral blood mononuclear cell (PBMC) of sepsis patients. These studies have identified several defective cellular pathways such as inhibited mitochondrial complex activity and oxygen consumption, and reduced ATP production across different sepsis populations [9,10,11]. However, these studies share a common limitation; they were conducted in patients with established sepsis or in the late stage of sepsis. Therefore, it is uncertain whether impaired cellular metabolism is present prior to the development of sepsis.

In this study, we sought to measure cellular metabolic state of PBMC in infection phase by studying peripheral blood samples from patients who present to emergency department with suspected infections, in whom sepsis is yet to develop. The overall goal of the study is to detect early changes in the metabolic profile of circulating immune cells in the infection patients and compare them to that of patients who later develop sepsis. In contrast, conventional biomarkers of infection and sepsis, such as C-reactive protein (CRP) and procalcitonin, are downstream markers that typically rise after sepsis has already developed, thereby limiting their utility as early indicators of patients at risk of progression to sepsis.

## Materials and methods

### Subjects Recruitment

This study was approved by Human Research Ethics Committee at our institution (reference number HREC/18/WMEAD/67). Patients were recruited from the emergency department (ED) at Westmead Hospital, New South Wales, Australia, between February 2018 and July 2019. Written informed consent was obtained from all subjects recruited. Inclusion criteria: subjects were eligible if they aged 18 years or older; presented to the ED within the last 24 hours with infection, defined as either (1) positive pathogen identification in any body fluids sampled for microbiological culture, or (2) a suspicion of infection (as determined by treating physician) and received antibiotics. Patients receiving ongoing medical treatment, including any form of immunosuppressive therapy, were eligible for inclusion. Exclusion criteria: (1) decision not to actively treat or resuscitate the patient at admission; and (2) inability to consent the patient.

### Case Definitions

**Fig. 1.**
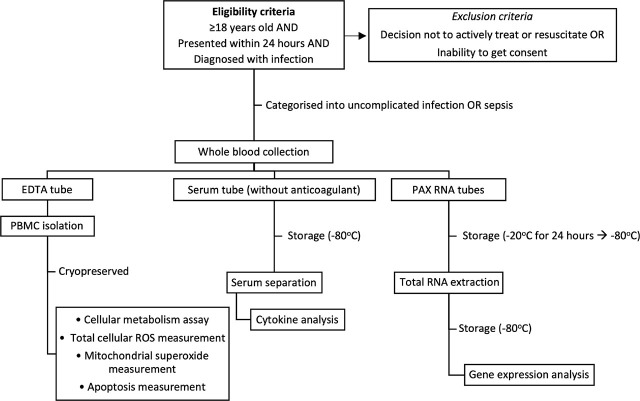
illustrates the workflow of experiment.

The study participants were assigned into sepsis or uncomplicated infection groups, based on their Sequential Organ Failure Assessment (SOFA) score on admission (≥2 and <2, respectively) in accordance with the international consensus definition of sepsis (“Sepsis-3”) [12, 13]. Healthy volunteers who did not have infection or any pre-existing comorbidities were recruited as controls (Figure 1).

### Gene Expression Profiling

#### RNA extraction

Two and a half millilitres of whole blood, which was collected into PAXgene Blood RNA tube (BD Biosciences, NJ, USA), was allowed to stand at room temperature for 2 hours, followed by storage at −20°C for 24 hours. Thereafter, the tubes were transferred to −80°C for long term storage. Total RNA was extracted using PAXgene Blood RNA Kit (QIAGEN, Manchester, UK) following the manufacturer’s protocol. Quantity and integrity of purified RNA were determined by the RNA integrity number (RIN) using TapeStation (Agilent Technologies Inc., CA, USA).

#### Gene expression by NanoString^(R)^ Technology

Gene expression was measured using Nanostring nCounter^®^ Elements™ TagSet (NanoString^(R)^ Technologies Inc., WA, USA) preselected for mitochondrial biogenesis and function panel. The panel includes 90 human genes and 6 housekeeping genes (Supplementary Table S1). One hundred nanograms of RNA from samples with RIN ≥7.0 was used for setting up the hybridization reaction. A standard nCounter Prep Station was set up on the workstation for post-hybridization processing. After which, sample cartridge was removed, sealed and loaded into the Digital Analyzer to be scanned. Data resolution was set to high (280 images per sample).

#### Gene expression data analyses

nCounter data files (RCC files) were imported into the nSolver™ analysis software v4.0 (NanoString Technologies, WA, USA) for quality control and raw mRNA abundance frequencies analysis. Quality control was performed with the built-in negative and positive controls following recommendations by NanoString Technologies. Raw data was normalized against the geometric mean of 6 housekeeping genes included in the codeset. The expression level was presented as log2-counts. Later on, to investigate whether the differentially expressed genes were associated with certain biological process, we performed enrichment analysis using MetaCore from Clarivate Analytics.

### Measurement of Serum Cytokines

Whole blood collected in tube without anticoagulant was allowed to clot for 30 minutes to 1 hour at room temperature before centrifugation at 1,600 × g for 15 minutes at room temperature. Supernatant was collected and stored at −80°C for further analyses.

Concentrations of cytokines were measured using the Bio-Plex Pro™ Human Cytokine Screening 6-Plex Panel (Bio-Rad Laboratories, CA, USA) according to the manufacturer’s protocol. Analytes measured were interleukin 1 beta (IL-1β), interleukin 4 (IL-4), interleukin 6 (IL-6), interleukin 10 (IL-10), tumour necrosis factor alpha (TNF-α) and interferon gamma (IFN-γ). Briefly, thawed serum samples were centrifuged at 10,000 × g for 10 minutes at 4°C to remove cells. Fifty microliters of the duplicated serial-diluted standards and serum samples were added to 96-well plate which contained the pre-washed antibody coated microbeads. The plate was sealed, followed by incubation at room temperature for 1 hour with shaking at 850 rotations per minute under dark. The plate was then washed 3 times with Bio-Plex Pro II magnetic plate washer and incubated with detection antibody for 30 minutes followed by 3 times washes and incubation with streptavidin-PE for 10 minutes at room temperature under dark. All the liquids were delivered to the assay plate using a robotic platform (epMotion 5075, Eppendorf). The plate was read with the Bio-Plex 200 system using the Bio-Plex manager v6.1 software.

### Measurement of Cellular Metabolism

Agilent Seahorse XF Analyser was used to measure two key parameters: oxygen consumption rate (OCR) and extracellular acidification rate (ECAR). These parameters reflect effectiveness of mitochondrial respiration (OCR) and glycolysis (ECAR), which provides an alternative energy pathway to mitochondrial respiration.

#### Sample collection and preparation

Whole blood sample was collected from healthy controls or study participants into tubes containing anticoagulant EDTA. Peripheral blood mononuclear cells were isolated from the whole blood using Ficoll. Isolated PBMCs were cryopreserved in freezing medium (fetal bovine serum (FBS) with 10% DMSO) and stored in Vapor Phase liquid nitrogen tank until further analyses.

#### Cellular metabolism assay

On the day of the assay, PBMCs were thawed and washed in RPMI + 10% FBS, and finally resuspended in Seahorse XF Assay Medium and seeded onto microplate pre-coated with Corning^(R)^ Cell-Tak Cell and Tissue Adhesive (Bio-Strategy Pty Ltd, NY, USA) at 5 × 10^5^ cells per well. Each sample was analysed at least in triplicate. Metabolic profiling of PBMC was assessed with the use of cell mito stress test kit (Agilent Technologies, Inc., CA, USA), which includes three drugs – oligomycin, carbonyl cyanide-4 (trifluoromethoxy) phenylhydrazone (FCCP) and rotenone/antimycin A. These drugs were pre-titrated and 2 μM oligomycin, 2 μM FCCP and 0.5 μM rotenone/antimycin A were used for our assays. Sequential injections of these drugs allowed the calculations of parameters related to mitochondrial respiration, including basal respiration, maximal respiration, spare respiratory capacity and ATP production [14].

Data was analysed using the Wave Desktop software version 2.6. To normalize the data with cell number, total protein amount was quantified at the completion of the XF assay using Bradford protein assay kit (Bio-Rad, CA, USA) after cells in the wells were lysed with lysis buffer (10 mM Tris, pH 7.4, 0.1% Triton X-100) [15].

### Measurement of Oxidative Stress

#### Total cellular reactive oxygen species (ROS)

To detect total cellular ROS, cells were stained with 2’,7’ – dichlorofluorescin diacetate (DCFDA) Cellular ROS Detection Assay Kit (abcam, Cambridge, UK) according to manufacturer’s protocol. Briefly, thawed PBMCs, resuspended in RPMI + 10% FBS, were allowed to rest for 2 hours before staining with 20 μM DCFDA for 30 minutes in 37°C CO_2_ incubator. Stained cells were subjected to analyses by BD FACs CantoII flow cytometer (BD Biosciences, CA, USA). Application setting was applied each time before analyses. Data was analysed using FlowJo v10 (BD Biosciences, Oregon, USA). Results were expressed as median fluorescence intensity (MFI) after subtracting background from unstained samples.

#### Mitochondrial superoxide

To quantify mitochondrial superoxide, cells were stained with MitoSOX™ Red (Invitrogen, MA, USA) according to manufacturer’s protocol. Briefly, thawed PBMCs were washed and resuspended in Hank’s Balanced Salt Solution with calcium and magnesium (HBSS/Ca/Mg), followed by staining with 5 μM MitoSOX™ Red for 10 minutes in 37°C CO_2_ incubator. After staining, cells were washed twice and resuspended in HBSS/Ca/Mg, which were then subjected to flow cytometry analyses as above.

### Measurement of Apoptosis

Cell apoptosis was quantified using Annexin V-FITC Apoptosis Detection Kit (abcam, Cambridge, UK) according to manufacturer’s protocol. Cells were washed and resuspended in 1x binding buffer, followed by staining with Annexin V-FITC and propidium iodide (PI). Stained cells were subjected to flow cytometry analyses as above. The Annexin V positive and PI negative cells were assigned as apoptotic cells.

### Statistical Analysis and Data Visualization

All statistical analyses were performed using SPSS version 25 (IBM) and GraphPad Prism 8. For the normally distributed data, analyses were conducted using parametric test. For data that was not normally distributed, data transformation was performed and was then subjected to parametric test. If the transformation was not successful to normalise the data, the non-parametric test was used. Additionally, multiple linear regression was used to identify confounder by assessing the change of p value and regression coefficient (≥10%) after the model was adjusted for potential confounder. Graphs were generated using GraphPad Prism 8. Heatmap was created using Morpheus software from Broad Institute (https://software.broadinstitute.org/morpheus).

## Results

### Patients Characteristics

Demographic and clinical characteristics of patients are summarized in Table 1. There was no significant difference in baseline characteristics between uncomplicated infection and sepsis groups except for age (p = 0.002) and number of patients with existing comorbidities (p = 0.015). The average age of sepsis group was higher than that of uncomplicated infection group (means 69 vs 56). Twenty healthy samples were recruited as controls, twelve (60%) of them were male. Their median age was 59 (28 – 72) years old.

**Table 1.** Patients Demographic and Clinical Characteristics (n = 67)

**Characteristics**	**Uncomplicated Infection**	**Sepsis**	**P value**
N (%)	27 (40.3)	40 (59.7)	
Age – yr	56.4 ± 18.12	69.1 ± 13.83	**0.002**
Male sex – no. (total no., %)	19 (70.4)	20 (50)	0.099

Source of infection			
Respiratory tract (%)	12 (44.4)	20 (50)	0.6551
Urinary tract (%)	4 (14.8)	12 (30)	0.1554
Abdominal, liver and biliary tract (%)	5 (18.5)	5 (12.5)	0.5022
Skin and soft tissue (%)	4 (14.8)	5 (12.5)	0.7881
Cardiovascular (%)	0 (0)	1 (2.5)	0.4113
Bone and joint (%)	1 (3.7)	1 (2.5)	0.7787
Unknown	2 (7.4)	1 (2.5)	0.3450

Comorbidities (%)	21 (77.8)	39 (97.5)	**0.0150**
Cardiovascular disease (%)	15 (55.6)	30 (75)	0.0997
Respiratory disease (%)	7 (25.9)	13 (32.5)	0.5654
Diabetes mellitus (%)	6 (22.2)	15 (37.5)	0.1887
Malignancy (%)	4 (14.8)	13 (32.5)	0.1050
Chronic kidney disease (%)	3 (11.1)	6 (15)	0.6485

Septic shock (%)	NA	10 (25.0)	NA
ICU admission (%)	0 (0)	9 (22.5)	**0.0086**
Hospital readmission – 28 day (%)	2 (7.4)	3 (7.5)	0.9879
Length of stay (day)	5 (1–68)	8 (1–106)	0.073
In-hospital mortality (%)	0 (0)	3 (7.5)	0.1484
Improving SOFA score on 3–5 days (%)	8/9 (88.9)	15/17 (88.2)	0.9585
Leukocyte count (×10^9^/L)	12.5 ± 3.72	14.6 ± 8.62	0.188
Neutrophil count (×10^9^/L)	10.0 ± 3.44	12.3 ± 8.17	0.124
Lymphocyte count (×10^9^/L)	1.3 (0.5–5.1)	0.9 (0.2–4.2)	0.053
Monocyte count (×10^9^/L)	0.7 (0.2–2.5)	0.9 (0.0–4.0)	0.453
Platelet (×10^9^/L)	222 (136–701)	187 (43–414)	**0.038**
CRP (mg/L)	43 (3–295)	118 (3–390)	**0.026**
Lactate (mmol/L)	1.45 (0.8–2.9)	2 (0.4–6.5)	**0.017**

Positive culture			
From source of infection (%)	11/24 (45.8)	20/37 (54.1)	0.5299
From blood (%)	6/21 (28.6)	13/32 (40.6)	0.3775

NA not available

Sepsis patients showed higher CRP and lactate levels as well as lower platelet count compared to the uncomplicated infection patients.

### Serum Cytokines’ Level Are Not Significantly Different Between Uncomplicated Infection and Sepsis Patients

Infection/sepsis was associated with changes in immune cell functions. We therefore sought to determine if the levels of serum cytokines can reflect changes in immune cell functions during the development of sepsis. For this experiment, we only analysed the data from 20 uncomplicated infection, 31 sepsis patients and 12 healthy controls. Data from the other samples were excluded as they were performed using different standards.

**Fig. 2.**
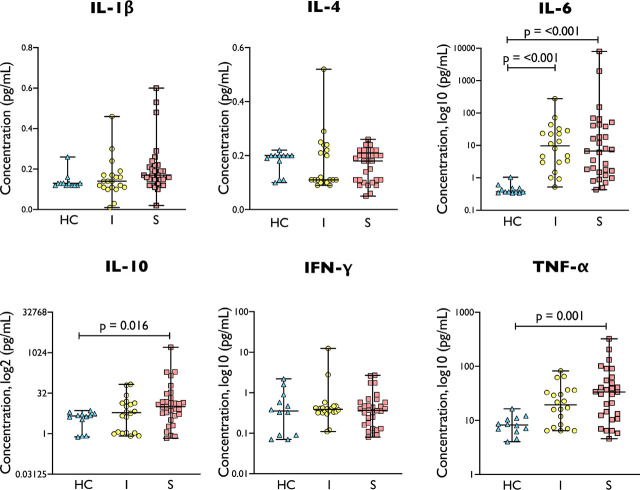
Cytokine levels in healthy control/HC (n = 12), uncomplicated infection/I (n = 20) and sepsis/S (n = 31). Sepsis demonstrated the highest cytokine levels in IL-6, IL-10 and TNF-α. Comparison between groups were performed by Kruskal-Wallis test followed by Dunn’s multiple comparison test.

In keeping with current literature [16], several inflammatory cytokines were higher in both uncomplicated infection and sepsis groups when compared to healthy controls (Figure 2). Serum levels of IL-10 and TNF-α were significantly higher in sepsis group than healthy controls but no difference was observed between sepsis and uncomplicated infection. Similarly, IL-6 level was higher in uncomplicated infection/sepsis patients compared to healthy controls but again no difference was observed between sepsis and uncomplicated infection.

### Downregulations of Mitochondrial Function-related Genes Are More Prominent in Sepsis Patients

Having found that inflammatory cytokines did not distinguish between uncomplicated infection and sepsis, we next proceeded to investigate whether gene-expression profiling could distinguish between such patients. As an initial explorative step, we performed gene expression profiling in the first 29 patients, as well as 11 healthy controls, recruited into the study (Supplementary Result, Table S2).

In these experiments, 29 out of a total of 90 genes showed differential expression (false discovery rate/FDR <0.05) across three groups – healthy controls, uncomplicated infection and sepsis groups (Figure 3A). From these 29 genes, 17 genes were significantly downregulated in infected/sepsis patients when compared to healthy controls (Figure 3B). Of these 17 genes, 13 genes were downregulated in the sepsis group but not in the uncomplicated infection groups. The remaining 4 genes were also downregulated in the uncomplicated infection. Twelve genes showed upregulation in infected/sepsis patients compared to healthy controls (Figure 3B). Of those genes, 5 genes were upregulated in uncomplicated infection as well as in sepsis group. The other 7 genes were upregulated only in sepsis group.

**Fig. 3A.**
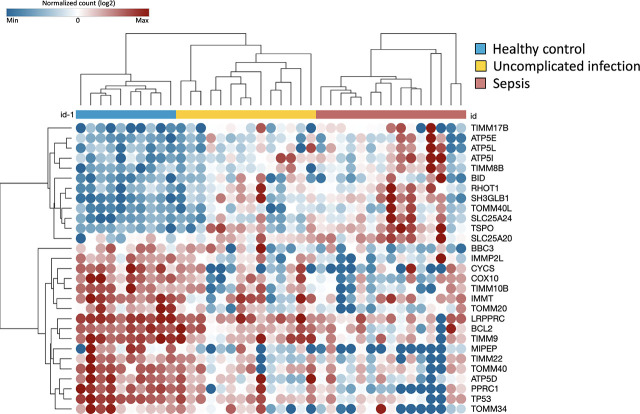
Heat map demonstrated 29 differentially expressed genes in log2-counts. The samples were grouped using semi-supervised clustering. Each sample is labelled by colour according to the clinical condition (performed on a subset of 10 healthy controls, 14 uncomplicated infection and 15 sepsis subjects).

**Fig. 3B.**
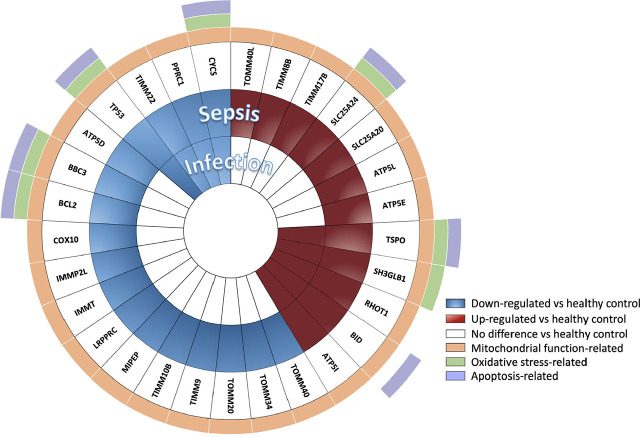
The differentially expressed genes (FDR <0.05) in uncomplicated infection (n = 14) and sepsis patients (n = 15) relative to healthy controls’ (n = 10). Function of each gene is indicated by colour (e.g. regulations on mitochondrial function, ROS production and apoptosis cell death). Twenty genes were expressed differently in sepsis. Comparison between groups were performed by one-way ANOVA followed by Benjamini-Yakutieli False Discovery Rate method.

Notably, there were 20 mitochondrial-related genes which were differentially expressed in those with sepsis but not in uncomplicated infection patients (Figure 3B). Enrichment analysis performed on those genes revealed that apoptosis regulation by mitochondrial protein (FDR 0.0028) and interleukin, those were IL-3 and IL-15, signaling (FDR <0.05) were amongst the significant pathways differentiating sepsis from uncomplicated infection (Supplementary Table S3). The genes involved in both pathways were BCL2 and BBC3. This finding points to a potential difference in leukocyte biology between uncomplicated infection and sepsis.

### Impaired Mitochondrial Function Is Observed in Uncomplicated Infection and Sepsis

**Fig. 4:**
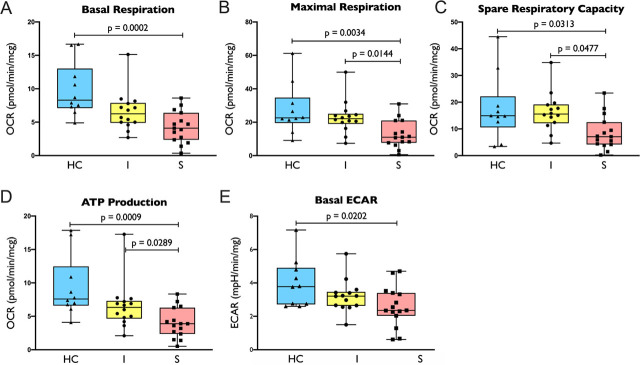
Oxygen consumption rate (OCR) and extracellular acidification rate (ECAR) on subjects with gene expression data: 4A. basal respiration, 4B. maximal respiration, 4C. spare capacity, 4D. ATP production, 4E. basal ECAR in healthy control/HC (n = 10), uncomplicated infection/I (n = 14) and sepsis/S (n = 15) group. Comparison between groups were performed by one-way ANOVA followed by Tukey’s multiple comparison test.

Having discovered the differences in gene expression related to mitochondrial pathway between uncomplicated infection and sepsis, we were to confirm it by measuring mitochondrial function of PBMC. Parameters related to mitochondrial functions including basal respiration, maximal respiration, spare respiratory capacity and ATP production reduced significantly in sepsis when compared to healthy control group (Figure 4 A–D). When the sepsis was compared to uncomplicated infection, maximal respiration, spare respiratory capacity and ATP production were significantly lower in sepsis group. Besides mitochondrial dysfunction, we also observed a reduced basal ECAR in sepsis when compared to healthy control (Figure 4E).

Having confirmed the evidence of impaired cellular metabolism in the above 29 patients, we next proceeded to measure the same metabolic parameters for the entire cohort (67 patients and 20 controls). These measurements again confirmed significant impairment and a trend towards impaired mitochondrial functions in sepsis and uncomplicated infection patients, respectively (Supplementary Result, Figure S1). The following findings described hereafter were performed on the entire cohort.

The above finding could be confounded by age since the sepsis group had a greater number of elderly patients compared to the uncomplicated infection group. Age *per se* is known to affect cellular metabolism [17]. To address this issue, we examined the association between infection severity (healthy controls, uncomplicated infection and sepsis groups) and mitochondrial function parameters with multiple linear regression adjusted for age. Notably, the association between infection severity and mitochondrial function parameters remained significant (adjusted p value <0.05), even though the regression coefficient was decreased by 8–14% after adjusted for age (Supplementary Table S4).

### Increased Mitochondrial Oxidative Stress Is Observed in Uncomplicated Infection and Sepsis Patients

**Figure 5:**
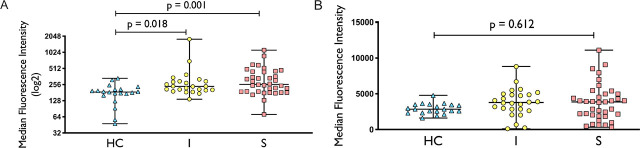
MitoSOX (5A) and DCFDA (5B) staining in healthy control (HC), uncomplicated infection (I) and sepsis (S). N for MitoSOX = HC (20), I (24), S (36), respectively; n for DCFDA = HC (20), I (26), S (37), respectively. For MitoSOX dataset, comparison between groups was performed with Kruskal-Wallis test followed by Dunn’s multiple comparison. For DCFDA dataset, comparison between groups was made using one-way ANOVA followed by Tukey’s multiple comparison.

Previous research shows that increased mitochondrial oxidative stress is associated with impaired mitochondrial function in circulating leukocytes [18]. Our results support this, as we observed increased mitochondrial superoxide level, as measured by MitoSOX, in both uncomplicated infection and sepsis groups when compared to healthy controls (Figure 5A). There was also a trend towards increased total cellular ROS (measured by DCFDA) in uncomplicated infection and sepsis when compared to healthy controls (Figure 5B) even though they were not statistically significant.

### Mitochondrial Oxidative Stress Correlates with Gene Expression and Serum Cytokine Level; and Associates with Bacteremia

Previous study showed that mitochondrial oxidative stress may be increased by several factors in infection/sepsis [19]. These factors include increased systemic inflammation (e.g. proinflammatory cytokines), pathogen factor (presence of bacteremia) and transcriptional factor (gene expression). To investigate the possible roles of those factors, we performed a correlation analysis between mitochondrial superoxide levels and: serum cytokine levels, presence of bacteremia and mitochondrial ROS-related genes including Cytochrome C/CYCS (electron transport chain), Tumor Protein P53/TP53 (maintains cellular redox status), Solute Carrier Family 25 Member 24/SLC25A24 (buffers calcium level in mitochondrial matrix) and Translocator Protein/TSPO (translocator protein for release of proapoptotic mitochondrial component).

Our analyses showed that mitochondrial superoxide level correlated with increased proinflammatory cytokine TNF-α (*r* = 0.330, p <0.05) but not with IL-6 and IL-10 (p >0.05). Similarly, mitochondrial superoxide level correlated with expression levels of CYCS, TP53, SLC24A24 and TSPO (*r*_s_ = −0.4926, −0.4422, 0.4382, 0.4835, respectively; p <0.05). Additionally, a higher oxidative stress level was observed in patients with compared to those without bacteremia (p <0.05) (Figure S2).

As to cellular metabolism, age is known to affect oxidative stress level [20]. To examine the effect of age, we performed linear regression by including age as independent variable in the association model between infection severity and mitochondrial superoxide level. The significant association (p <0.05) between infection severity and mitochondrial superoxide level became non-significant (p ≥0.05) after age was included in the model. The regression coefficient was also reduced by ≥10% (Supplementary Table S4). Therefore, the association between infection severity and mitochondrial superoxide level was confounded by age.

### Impact of Increased Oxidative Stress on Leukocyte Function and Survival

**Fig. 6.**
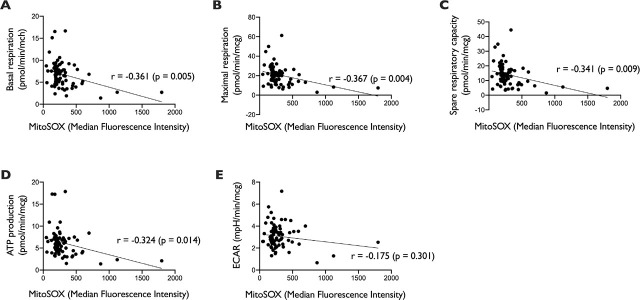
Correlation between mitochondrial superoxide level (measured with MitoSOX) with cellular metabolism parameters (n = 80) (A. Basal respiration, B. Maximal respiration, C. Spare respiratory capacity, D. ATP production, E. ECAR). Analysis was performed with Pearson correlation. r = correlation coefficient.

Even though increased levels of mitochondrial superoxide was not significantly associated with infection severity, we would like to evaluate the association of this increase with leukocyte mitochondrial functions. Our analyses revealed a significant inverse correlation between an increased mitochondrial superoxide level and mitochondrial function (p <0.05) (Figure 6). The findings were consistent across all parameters as measured by our cellular metabolism assay. To examine the effect of age on the correlation, we performed partial correlation analysis between mitochondrial superoxide level and mitochondrial function parameters by including age as controlling factor. The correlations remained significant between those variables:

$$\begin{array}{c} {\text{r}}_{\text{basal}\;\text{respiration}}=-0.309,\text{p}=0.006;\\ {\text{r}}_{\text{maximal}\;\text{respiration}}=-0.318,\text{p}=0.004;\\ {\text{r}}_{\text{spare}\;\text{respiratory}\;\text{capacity}}=-0.295,\text{p}=0.008;\\ {\text{r}}_{\text{ATP}\;\text{production}}=-0.269,\text{p}=0.016. \end{array}$$

Existing literature shows both impaired mitochondrial function and increased intra-mitochondrial oxidative stress may lead to the activation of apoptotic pathways [21]. However, we did not observe any significant increase in apoptosis measured by Annexin V and PI in PBMCs from either the uncomplicated infection or the sepsis group (Figure S3).

## Discussions

Currently, it remains unclear whether defective cellular metabolism exists in circulating leukocytes of patients who are in the early stage sepsis. Our study provide evidence that the defect is present in patients who are infected and more so in those who progress to sepsis. The impaired cellular metabolism is evident across several platforms including gene-expression profiling, cellular metabolism analyses and intra-mitochondrial reactive oxygen species measurement.

In the cohort subset, the mitochondrial function was significantly reduced in sepsis compared to the uncomplicated infection patients. However, statistical difference on mitochondrial function was not detected from the analysis of entire cohort, although a trend toward it was observed. Amongst the most possible explanations were the age difference between uncomplicated infection and sepsis patients in which the difference was wider in the subset (51.07 vs. 73.27, respectively. Data was not presented) than in the entire cohort (56.4 vs. 69.1, respectively).

Our finding of worse mitochondrial function impairment in sepsis was supported by the finding of BCL2 and BBC3 downregulation. The latter finding was detected in sepsis but not in uncomplicated infection. Downregulation of BCL2 has been associated with the loss of mitochondrial membrane potential that leads to apoptosis [22]. As of BCL2, transcriptional regulation of BBC3 has been linked to mitochondrial events associated with apoptosis [23]. Our analysis also showed that those genes regulated IL-3 and IL-15 pathways. Both interleukins are acknowledged to amplify inflammatory response in sepsis [24, 25]. As discussed earlier, proinflammation in sepsis potentially affects the oxidative stress level as well as mitochondrial function. Altogether, those findings may explain the more severe impairment of mitochondrial function in sepsis.

Although we do not have direct evidence to implicate oxidative stress as a causal factor in the impairment of cellular metabolism, we do observe a moderate correlation between the degree of mitochondrial oxidative stress and the extent of cellular respiration impairment, i.e. increasing intra-mitochondrial oxidative stress correlates with worsening of cellular respiration. The increase in intra-mitochondrial oxidative stress level, apart from the confounding effect of age, is associated with higher proinflammatory cytokine, bacteremia and gene dysregulation. Inflammatory cytokine and dysregulation of genes are known to be associated with oxidative stress and, in general, reduced cellular respiration [26, 27]. The presence of bacteremia, as shown in this study, is also associated with increased oxidative stress since bacterial infection is the trigger of ROS production [28].

The relationship between proinflammatory cytokine, oxidative stress and impaired mitochondrial function is complex. The levels of proinflammatory cytokines significantly show correlation with oxidative stress and impaired mitochondrial function. Mitochondrial oxidative stress itself can further activate inflammatory response through the production of signalling molecules that propagate cellular dysfunction [29]. Therefore, it is likely that changes in systemic inflammation milieu, intra-mitochondrial oxidative stress and cellular respiration are intricately linked. It is beyond the scope of our study to delineate such complex relationship; clearly, additional mechanistic studies are needed in the future.

We also observe that in uncomplicated infection patients, the circulating immune cells do not shift the ATP production to glycolysis. Existing literatures indicate that host cells typically switch their main metabolism into glycolysis when stressed [30, 31]. The lack of such a switch might be because immune cells may utilize pathways alternative to glycolysis to generate more ATP. Additionally, different immune cell subsets prefer to use different dominant type of metabolism, e.g. naïve T cells are dependent on mitochondrial respiration as their primary metabolic pathway while activated T cells exhibit higher glycolysis [32]. Further studies are needed to address this issue, as by using glycolysis-specific assay in purified cell subsets.

This study demonstrates the lack of cytokine use as a marker for staging sepsis patients [33]. The increase of cytokines found in this study could not distinguish sepsis from the uncomplicated infection, on which the routine laboratories, such as CRP, lactate level and platelet count, could discriminate those severities better. This indicates the need to develop more specific yet practical markers that enable us to stage infection patients according to their risk severities.

Our findings have several clinically relevant implications that warrant further investigation. The presence of increased mitochondrial oxidative stress in PBMCs during uncomplicated infection suggests that immune cell metabolic perturbations occur early in the disease course, preceding overt clinical deterioration. Previous studies have demonstrated that mitochondrial dysfunction is a key feature of established sepsis and is associated with immune paralysis, organ dysfunction, and adverse outcomes. Our observations extend these findings by suggesting that mitochondrial oxidative stress may represent an upstream event that contributes to subsequent mitochondrial impairment as infection progresses. If this temporal relationship is confirmed, early alterations in immune cell metabolism could provide a window for identifying patients who are biologically vulnerable to progression despite appearing clinically stable. In this context, simplified surrogate markers—such as transcriptional signatures related to mitochondrial stress or circulating inflammatory–metabolic profiles—may have clinical utility as adjunctive tools for early risk stratification, complementing existing clinical scores that rely primarily on physiological and inflammatory parameters.

We have used multiple modalities from transcriptomic to functional analyses to investigate the change in mitochondrial and immune function which has strengthened this research. Additionally, this study included infection patients from ED instead of the “established” sepsis from intensive care unit (ICU). Therefore, we could observe metabolic changes in broader spectrum of disease rather than simply comparing the changes between severe disease and the normal end. In regard to it, the finding of impaired cellular metabolism in uncomplicated infection consequently suggests that past biomarkers developed in the critically ill, i.e. ICU, patients might have low relevance in ED setting.

Some limitations are notified in this study. First, the study participants are highly heterogenous in comorbidities and age. Those factors, particulary age, should be put into consideration when interpreting the result, i.e. patients with diabetes mellitus or older age might have different basal oxidative stress level. Secondly, most of the study participants have favorable outcome, i.e. minimal number of mortality. In the same manner, the analysis is ideally explored in patients with poor outcome, as the result might be variable from those with good prognosis. Thirdly, total ROS is not an ideal assay to measure oxidative stress since, technically, intermediate radical will falsely amplify the fluorescence intensity [34, 35]. The non-significant signal of total cellular ROS might be explained by this issue. Lastly, this was a single-centre study with a relatively small sample size compared with other sepsis cohorts; therefore, the generalisability of the findings to broader population should be interpreted with caution.

Surprisingly, we did not observe increased cell death in this study, despite significant increase in mitochondrial oxidative stress levels and impairment of mitochondrial function. Although no increase in overt apoptosis was detected, several early and upstream markers of apoptotic regulation—including altered BCL-2 family gene expression, CYCS and TP53 transcription—were observed, suggesting apoptotic priming rather than execution. Another possible explanation could be that dying cells are not captured during sample processing procedure. To address this issue, early marker of apoptosis can be used in future study, such as caspase activation and mitochondrial membrane potential [36].

## Conclusions

In conclusion, impairment of immune cell metabolism, presented mainly as mitochondrial dysfunction, is already evident in patients with infection who do not fulfil Sepsis-3 criteria. This mitochondrial dysfunction is associated with increased intramitochondrial oxidative stress in both uncomplicated infection and established sepsis, although this association appears to be partially confounded by age. Intramitochondrial oxidative stress also correlates with pro-inflammatory signaling and the transcriptional regulation of apoptosis-related pathways. Collectively, these findings suggest that metabolic and mitochondrial perturbations in circulating immune cells occur early during infection and may precede clinical progression to sepsis.
